# Magnetic Resonance Imaging Visualizes Median Nerve Entrapment due to Radius Fracture and Allows Immediate Surgical Release

**DOI:** 10.1155/2015/703790

**Published:** 2015-01-01

**Authors:** Satoshi Yanagibayashi, Naoto Yamamoto, Ryuichi Yoshida, Mitsuru Sekido

**Affiliations:** ^1^Department of Plastic and Reconstructive Surgery, New Tokyo Hospital, 1271 Wanagaya, Matsudo, Chiba 270-2232, Japan; ^2^Department of Plastic and Reconstructive Surgery, Institute of Clinical Medicine, University of Tsukuba, Ibaraki 305-0005, Japan

## Abstract

Median nerve entrapment with forearm fracture is rare, and surgical exploration in the early stage is rarely performed. We report the case of a 19-year-old man presenting with severe pain and numbness of the thumb, index, and middle fingers and half of the ring finger along with weakness of abduction and opposition of the thumb after fracture of the radial shaft. These symptoms remained unimproved despite precise closed reduction and cast immobilization. The radius fracture was barely displaced, but complaints were increasing, particularly when the wrist and/or fingers were stretched. This suggested direct involvement of the median nerve at the fracture site, so magnetic resonance imaging (MRI) of the forearm was performed to identify any entrapment. Short tau inversion recovery MRI visualized significant deviation and entrapment of the median nerve at the fracture site. Surgical release of the entrapment was performed immediately, and complaints resolved shortly thereafter. A positive Tinel sign from the palm to the fingertips and recovery of abduction and opposition of the thumb were seen at 6 months postoperatively. This report highlights the utility of MRI for detecting median nerve entrapment at a fracture site, allowing immediate surgical release.

## 1. Introduction

Closed forearm fracture sometimes leads to complaints of severe pain, numbness, and/or weakness of the muscle in the territory of median nerve innervation. These symptoms are often transient and attributable to stretching of the median nerve near the fracture site. However, symptoms in some cases persist for several months, with median nerve entrapment only found after bone union has been established [1–11]. The reason for the delay in diagnosis is that median nerve entrapment is only suspected based on indirect evidence such as clinical findings and nerve conduction velocity. More reliable and direct evidence is thus needed before surgical exploration in the early stage of median nerve complaints.

We encountered a case with median nerve entrapment associated with a simple radius shaft fracture. Magnetic resonance imaging (MRI) clearly depicted the entrapment at the fracture site and surgical release was immediately and successfully performed.

## 2. Case Presentation

**Figure 1:**
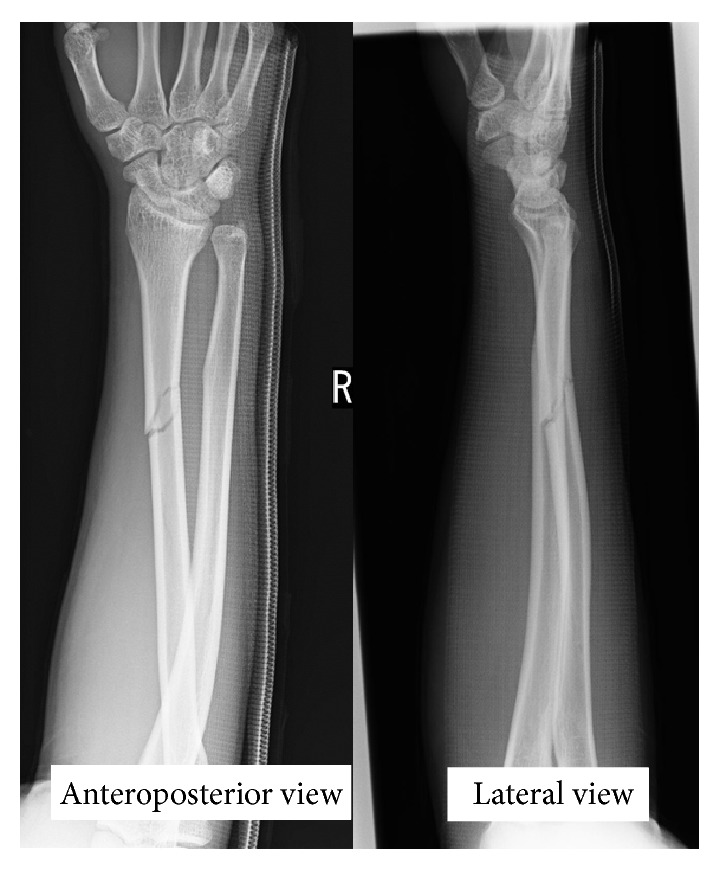
Radiograph of the fractured radius on admission.

**Figure 2:**
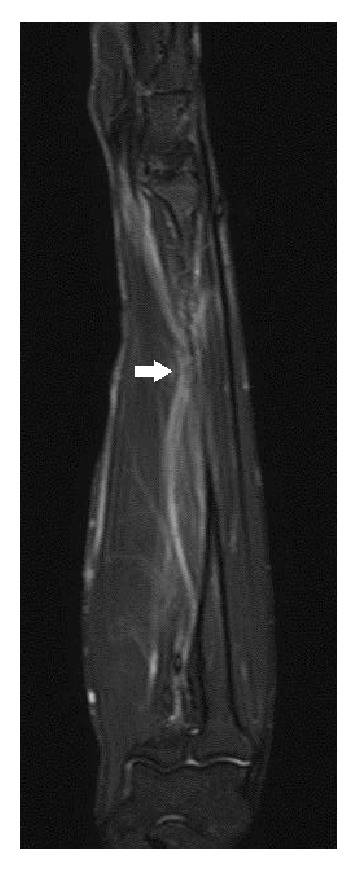
Short tau inversion recovery magnetic resonance imaging shows significant deviation of the median nerve toward the fracture site of the radius (arrow).

**Figure 3:**
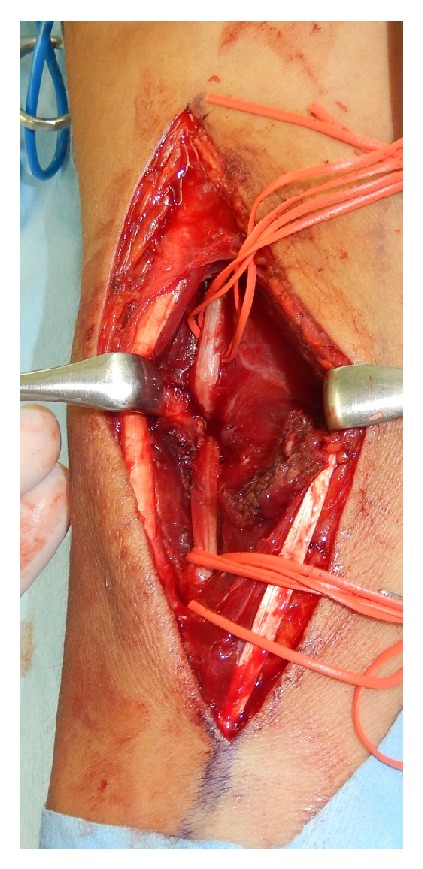
Intraoperative view of the entrapped median nerve at the fracture site of the radius.

A 19-year-old man fell while playing football, and a closed, slightly displaced fracture of the right radius was identified. He underwent closed reduction and cast immobilization at a local hospital (Figure 1). After reduction, he complained of persisting severe pain and numbness of the thumb, index, and middle fingers and half of the ring finger. The next day, he attended our hospital. Despite rest and elevation of the forearm and intravenous administration of steroids for 6 days, symptoms remained unimproved. When stretching the right fingers and wrist at the time of cast change, the severity of symptoms increased and weakness of the abductor pollicis brevis and opponens pollicis muscles was observed. Median nerve entrapment at the fracture site was therefore suspected, and MRI was performed on day 7 after injury to depict the median nerve in the forearm and clarify the indications for surgical exploration. MRI using the short tau inversion recovery (STIR) technique revealed significant deviation and entrapment of the median nerve at the fracture site (Figure 2). Almost all of the median nerve was trapped within the fracture site, but release was successfully achieved with a surgical procedure on day 10 after injury (Figure 3). The median nerve was constricted at the site of entrapment, but continuity was maintained. Immediately postoperatively, the patient reported improvement of symptoms. By 6 months postoperatively, a positive Tinel sign was identified from the palm to the fingertips and recovery of abduction and opposition of the thumb was also observed.

## 3. Discussion

Median nerve entrapment in association with forearm fractures is uncommon. We identified eleven other cases of median nerve entrapment associated with forearm fracture [1–11]. With the exception of one case, these previously reported entrapments were only released or repaired surgically after bone union had already been established. The remaining case was explored and an entrapped nerve was freed in the acute setting after clinical findings suggested median nerve injury or developing compartment syndrome [9]. The present report represents the first description of median nerve entrapment with radius fracture alone, as all the other cases involved either fracture of both the radius and ulna or ulnar fracture alone.

Generally, closed reductions are selected for simple mid-shaft forearm fracture, even if symptoms showing a median nerve distribution are reported. Surgical exploration to search for median nerve entrapment is thus rarely performed in the acute phase. However, symptoms of median nerve dysfunction sometimes persist for several weeks or months despite precise reduction and casting. At this time, belated identification of entrapment and release of the entrapped median nerve are performed. Previously reported cases of median nerve entrapment were released after 39 days–24 months [1–11]. Reasons for such delayed diagnosis include the unclear nature of complaints from affected children and the assumption that such numbness will prove to be temporary. If surgical exploration is performed after several months, neurolysis and release of the entrapped median nerve may prove to be more difficult and complicated than that before bone union, because bone union at the fracture site has been established to involve the median nerve. However, previously reported median nerve entrapments still showed satisfactory progression of improvement after release of the entrapped nerve. A wait-and-see attitude can be adopted on the expectation of neurapraxia with closed forearm fracture, but to minimize the duration of disease, diagnosis and treatment of median nerve entrapment should still be performed as soon as possible if entrapment is suspected.

Median nerve entrapment at the fracture site should be strongly suspected when a patient describes nonimproving numbness and pain showing distribution in the territory of the median nerve after closed reduction of a forearm fracture, particularly on stretching of the fingers or wrist, which stretches the entrapped median nerve. However, clinicians are rightfully hesitant to perform invasive procedures such as exploration for entrapped median nerve without more reliable evidence. Therefore, if median nerve entrapment is suspected, visualization of the median nerve on MRI is warranted for precise and early diagnosis. Fat-suppression techniques such as STIR will clearly show the course of the median nerve [12], with entrapment depicted as a deviation toward the fracture site.

Yeo et al. reported median nerve entrapment visualized with MRI after bone union had already been established [11], but our case represents the first report of an entrapped median nerve visualized on MRI before bone union. Entrapment neuropathy [12], especially in median nerve mononeuropathy [13], may not be accurately demonstrated on MRI, but the modality remains useful for tracing the course of median nerve in forearm fractures [11].

When median nerve entrapment with forearm fracture is suspected, MRI allows visualization of the entrapment for immediate and precise diagnosis, and surgical exploration can immediately be performed to release the entrapment.
